# Pretreatment with High Mobility Group Box-1 Monoclonal Antibody Prevents the Onset of Trigeminal Neuropathy in Mice with a Distal Infraorbital Nerve Chronic Constriction Injury

**DOI:** 10.3390/molecules26072035

**Published:** 2021-04-02

**Authors:** Takahiro Kochi, Yoki Nakamura, Simeng Ma, Kazue Hisaoka-Nakashima, Dengli Wang, Keyue Liu, Hidenori Wake, Masahiro Nishibori, Masahiro Irifune, Norimitsu Morioka

**Affiliations:** 1 Department of Pharmacology, Graduate School of Biomedical and Health Sciences, Hiroshima University, 1-2-3 Kasumi, Minami-ku, Hiroshima 734-8553, Japan; d173740@hiroshima-u.ac.jp (T.K.); nakayoki@hiroshima-u.ac.jp (Y.N.); m202697@hiroshima-u.ac.jp (S.M.); hisaokak@hiroshima-u.ac.jp (K.H.-N.); 2 Department of Dental Anesthesiology, Graduate School of Biomedical and Health Sciences, Hiroshima University, 1-2-3 Kasumi, Minami-ku, Hiroshima 734-8553, Japan; mirifun@hiroshima-u.ac.jp; 3 Department of Pharmacology, Graduate School of Medicine, Dentistry and Pharmaceutical Sciences, Okayama University, 2-5-1 Shikata-cho, Okayama 700-8558, Japan; dengliwang@md.okayama-u.ac.jp (D.W.); liukeyue@md.okayama-u.ac.jp (K.L.); wake-h@cc.okayama-u.ac.jp (H.W.); mbori@md.okayama-u.ac.jp (M.N.)

**Keywords:** pain, microglia, trigeminal neuropathy, macrophage, high mobility group box 1, neutralizing antibody

## Abstract

Persistent pain following orofacial surgery is not uncommon. High mobility group box 1 (HMGB1), an alarmin, is released by peripheral immune cells following nerve injury and could be related to pain associated with trigeminal nerve injury. Distal infraorbital nerve chronic constriction injury (dIoN-CCI) evokes pain-related behaviors including increased facial grooming and hyper-responsiveness to acetone (cutaneous cooling) after dIoN-CCI surgery in mice. In addition, dIoN-CCI mice developed conditioned place preference to mirogabalin, suggesting increased neuropathic pain-related aversion. Treatment of the infraorbital nerve with neutralizing antibody HMGB1 (anti-HMGB1 nAb) before dIoN-CCI prevented both facial grooming and hyper-responsiveness to cooling. Pretreatment with anti-HMGB1 nAb also blocked immune cell activation associated with trigeminal nerve injury including the accumulation of macrophage around the injured IoN and increased microglia activation in the ipsilateral spinal trigeminal nucleus caudalis. The current findings demonstrated that blocking of HMGB1 prior to nerve injury prevents the onset of pain-related behaviors, possibly through blocking the activation of immune cells associated with the nerve injury, both within the CNS and on peripheral nerves. The current findings further suggest that blocking HMGB1 before tissue injury could be a novel strategy to prevent the induction of chronic pain following orofacial surgeries.

## 1. Introduction

Posttraumatic trigeminal neuropathy (PTTN) is a chronic sensory disorder characterized by chronic orofacial numbness, paresthesia and pain [1]. Around 3–7% of orofacial procedures, such as injection of local anesthesia, implant surgery, and third molar removal, result in PTTN [1]. Pain associated with PTTN is refractory to commonly used analgesics such as opioids and non-steroidal anti-inflammatory drugs [2]. It is believed that the risk of developing PTTN increases with the complexity of the surgical procedure [3]. Additionally, unlike other painful peripheral neuropathies, the potential of developing PTTN can be predicted based on preoperative assessment, such as X-ray of the trigeminal nerve tract or of the surgery site. Although a preoperative strategy reduces the potential for pain following PTTN, pain occurs following PTTN despite careful control. Therefore, the molecular mechanism of pain following PTTN has to be clarified.

High mobility group box-1 (HMGB1) is a non-histone DNA binding protein involved in the regulation of gene transcription and replication. It is abundantly found in the cell nuclei of most mammalian cells [4]. High mobility group box-1 passively diffuses from damaged cells and is also actively secreted from immune cells in the form of an alarmin/damage-associated molecular patterns (DAMPs) [5]. In the CNS, extracellular HMGB1 binds to a number of receptors, including toll-like receptor (TLR) 4 receptors, receptor for advanced glycation end-products (RAGE) and C-X-C chemokine receptor type 4 (CXCR4) and binding to these receptors leads to inflammation, which may be an important mechanism of neurological disorders such as Alzheimer’s diseases, Parkinson’s disease and stroke [6]. In the peripheral nervous system, previous studies have demonstrated that HMGB1 diffuses from the site of a nerve injury and plays a crucial role in maintaining the neuropathic pain state [7,8,9,10]. It is possible that HMGB1 leaked from injured trigeminal nerves or damaged Schwann cells surrounding trigeminal nerves is involved in the induction and maintenance of PTTN though a localized inflammatory response. Thus, blocking HMGB1 before trigeminal nerve injury could prevent the onset of PTTN pain.

Previous studies have demonstrated that activation of peripheral and central immune cells is critical in the onset and maintenance of chronic neuropathic pain [11]. Macrophage accumulates around the sciatic nerve following a nerve injury and the extent of macrophage accumulation is associated with cutaneous hypersensitivity [12]. Trigeminal nerve injury induces robust microglial activation in the ipsilateral spinal trigeminal nucleus caudalis (Sp5C) [13,14]. Microglial activation in other brain regions has been associated with cutaneous hypersensitivity [15]. Thus, it is possible that peripheral and central immune cell activation could be utilized as a cellular, non-neural markers of pain following nerve injury.

The current study sought to define the involvement of HMGB1 in pain evoked in a mouse model of PTTN by examining the effect of early blockade of HMGB1 on pain-related behaviors using an HMGB1 neutralizing antibody (anti-HMGB1 nAb). As blocking HMGB1 reduces immune cell responses, the effect of anti-HMGB1 nAb treatment on macrophage accumulation around the injured infraorbital nerve (IoN) and microglial activation in the Sp5C were also examined. The current findings suggest early blockade of the inflammatory response to trigeminal neve injury prevents PTTN pain.

## 2. Results

### 2.1. Distal Infraorbital Nerve Chronic Constriction Injury Induces Long-Lasting Orofacial Pain Behavior

**Figure 1:**
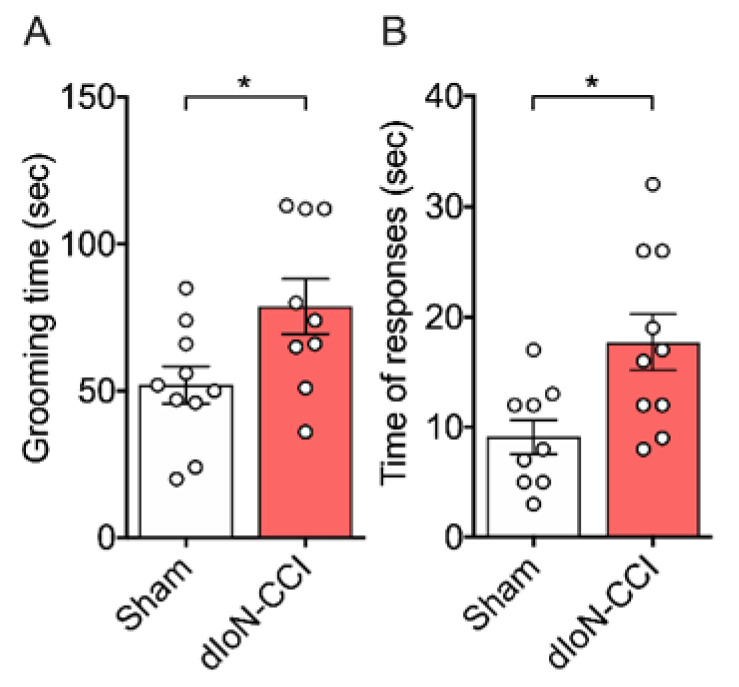
Pain-related behaviors following distal infraorbital nerve chronic constriction injury (dIoN-CCI) in mice. (**A**) Total grooming time in sec. (*n* = 9–10/group) and (**B**) total number of responses to acetone (*n* = 9–10/group) were assessed 7 and 13 days after the surgery, respectively. Individual data and mean ± SEM are shown. * *p* < 0.05 compared to sham (unpaired *t* test).

Total facial grooming time was significantly greater in distal infraorbital nerve chronic constriction injury (dIoN-CCI) mice compared to that of sham mice (Figure 1A, Unpaired *t* test, Table S1), suggesting dIoN-CCI-induced spontaneous pain. Increased wiping behavior following acetone application was observed in dIoN-CCI mice compared to that of sham mice, suggesting cooling hypersensitivity following dIoN-CCI (Figure 1B, Unpaired *t* test, Table S1). Placement of 15 µL of room temperature water on the ipsilateral face of either sham or dIoN-CCI resulted in no (or <1 s) wiping behavior (data not shown).

### 2.2. Conditioned Place Preference to Mirogabalin Following dIoN-CCI

**Figure 2:**
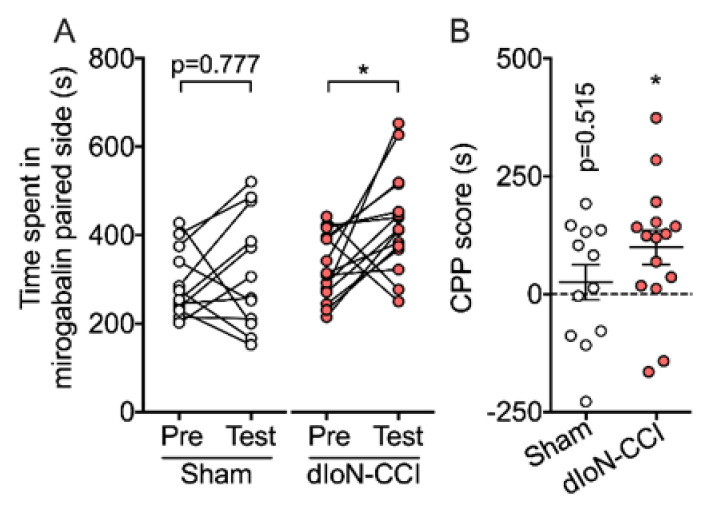
Conditioned place preference (CPP) in sham and IoN-CCI mice. Mirogabalin (2 mg/kg, i.p.) was injected to each mouse and placed in CPP chamber. (**A**) Total time spent in the mirogabalin-paired compartment during the pre-conditioning (pre) and post-conditioning (post) test sessions. Sham (*n* = 12) and dIoN-CCI (*n* = 15). Mice with a dIoN-CCI developed a preference for the mirogabalin-paired compartment, whereas sham mice showed no preference. Individual data and mean ± SEM are shown. * *p* < 0.05 compared with pre-conditioning (two-way RM ANOVA followed by Sidak’s multiple comparisons test). (**B**) total CPP scores. sham (*n* = 12) and dIoN-CCI (*n* = 15). Data are expressed as mean ± SEM. * *p* < 0.05 (One sample *t*-test).

Significantly longer times were spent in the mirogabalin-paired side compared to that during the pretest session in dIoN-CCI mice, whereas no significant change in time spent in the mirogabalin-paired was observed in sham-operated mice. (Figure 2A; Two-way RM ANOVA, Sidak’s multiple comparisons test; Table S1). Moreover, in mirogabalin-treated dIoN-CCI mice, conditioned place preference (CPP) score was significantly increased compared with their baseline CCP score (0) (Figure 2B, one-sample *t* test, Table S1). In contrast, treatment of sham-operated mice with mirogabalin did not increase CPP scores (Figure 2B, one-sample *t* test, Table S1).

### 2.3. Macrophage Accumulation around the IoN Following dIoN-CCI

**Figure 3:**
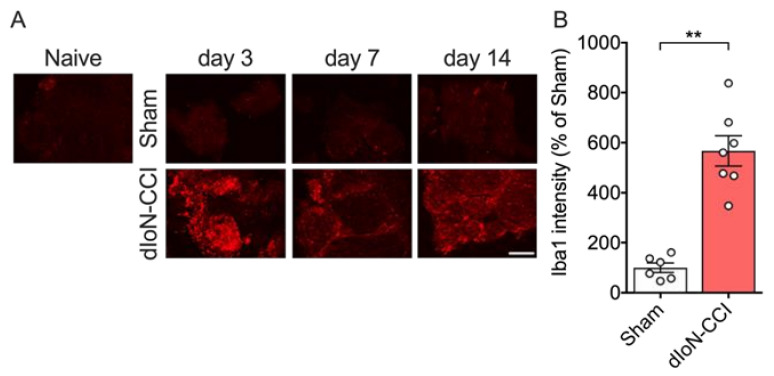
Macrophage accumulation around infraorbital nerve (IoN) following sham and dIoN-CCI. (**A**) Fluorescent photomicrographs of Iba1, a macrophage marker, of IoN from naïve, sham-operated and dIoN-CCI mice 3, 7, 14 days following surgery. One section was taken from one mouse on Day 3 and Day 7. One section was taken from seven mice on Day 14. Scale bar = 50 μm. Mean percent Iba1 intensity (percent of sham) (**B**). Data are expressed as individual and mean ± SEM. *n* = 6–7. ** *p* < 0.01 compared with sham-operated (unpaired *t*-test).

Macrophage accumulation (Figure 3A) and Iba1 immunoreactive cells around the ipsilateral IoN of dIoN-CCI (Figure 3B) were observed beginning 3 days after dIoN-CCI surgery and sustained for at least 14 days after surgery (unpaired *t* test, Table S1). No macrophage accumulation was observed in sham-operated nerves at any time after sham surgery.

### 2.4. Microglial Activation in Sp5C Following dIoN-CCI

**Figure 4:**
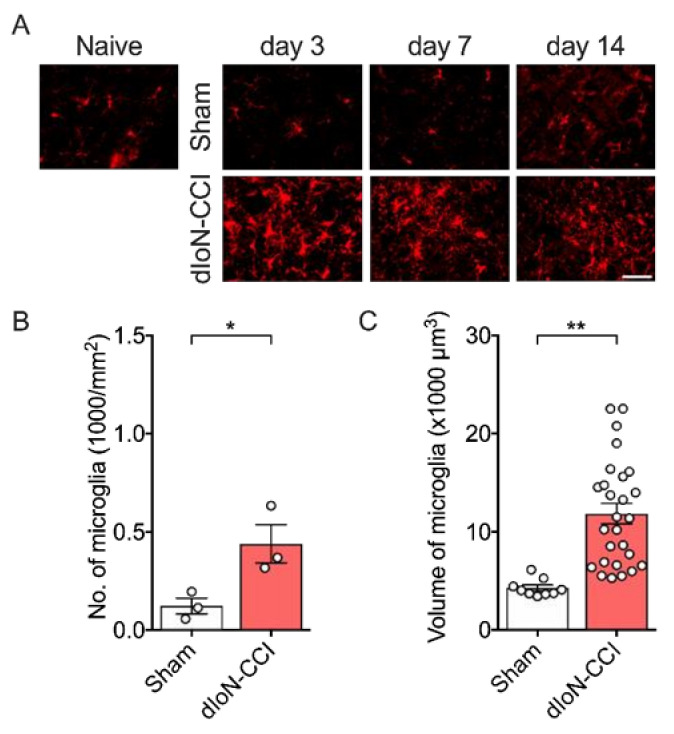
Activation of Sp5c microglia following sham and dIoN-CCI. (**A**) Fluorescent photomicrographs of Iba1, a microglial marker, Sp5C from naïve, sham-operated and dIoN-CCI mice 3, 7, 14 days following surgery. One section was taken from one mouse on Day 3 and Day 7. One slide was taken from seven mice on Day 14. Scale bar = 50 μm. (**B**) Mean density (number of cells/mm^2^) of *n* = 3 mice and (**C**) individual cell volumes (*n* **=** 9 (sham) or 27 (dIoN-CCI) cells from 3 consecutive ipsilateral sections) in Sp5C 14 days following surgery. Data are expressed as individual and mean ± SEM. * *p* < 0.05, ** *p* < 0.01 compared with sham-operated (unpaired *t*-test).

Both the number of microglia and cell volume ipsilateral to dIoN-CCI were increased in ipsilateral Sp5C 3 days and 14 days after surgery (Figure 4A,B, unpaired *t* test, Table S1; Figure 4C, unpaired *t* test, Table S1). By contrast, sham surgery did not lead to increased microglia activation in the ipsilateral Sp5C at any time point.

### 2.5. Early Perineural Pretreatments with Anti-HMGB1 nAb Blocks Pain-Related Behaviors and Microglial Activation Following dIoN-CCI

#### 2.5.1. Orofacial Pain Behavior

**Figure 5:**
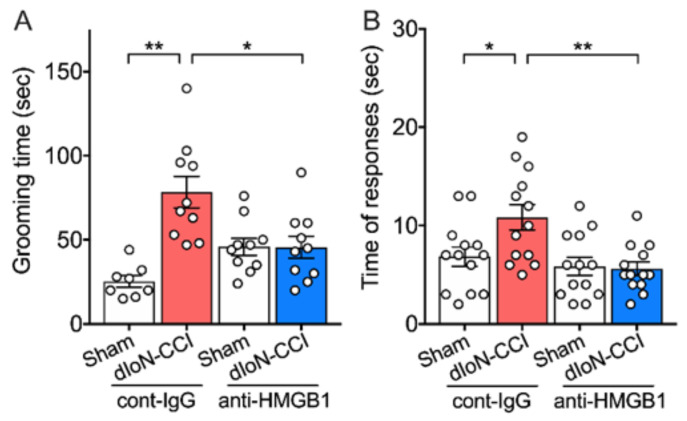
Effect of anti-HMGB1 nAb treatment on pain-related behavior in dIoN-CCI mice. (**A**) Total facial grooming time (*n* = 8–10 per group) and (**B**) total response time to acetone (*n* = 13 per group) were assessed 7 and 13 days after the surgery, respectively. Data are expressed as individual and mean ± SEM. * *p* < 0.05, ** *p* < 0.01 compared with sham-operated (Two-way ANOVA, Sidak’s correction for multiple comparisons).

Increased facial grooming time and hypersensitivity to cooling following dIoN-CCI were prevented with early perineural pretreatments with anti-HMGB1 nAb (Figure 5A,B; Two-way ANOVA, Sidak’s multiple comparisons test; Table S1). In contrast, treatment with anti-HMGB1 nAb 7 and 9 days after dIoN-surgery did not affect dIoN-CCI-evoked responses to acetone (Figure S1; Unpaired *t* test, Table S1).

#### 2.5.2. Macrophage Accumulation

**Figure 6:**
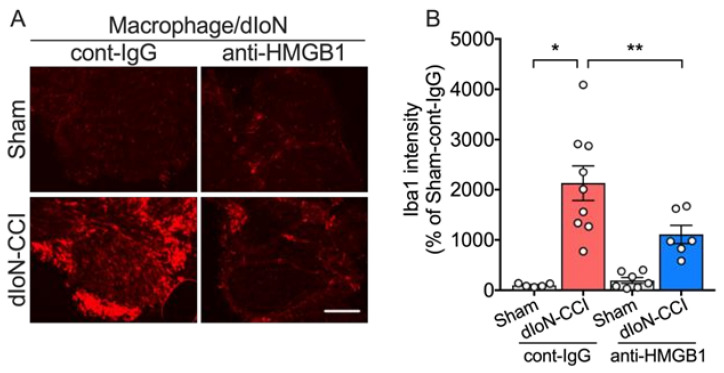
Effect of neutralizing antibody HMGB1 (anti-HMGB1 nAb) on macrophage accumulation in sham and dIoN-CCI mice. (**A**) Fluorescent photomicrographs of Iba1, a macrophage marker, of IoN from sham and dIoN-CCI mice 14 days following surgery. (**B**) Mean Iba1 intensity (*n* = 5–9 mice). One section was taken from one mouse on Day 3 and Day 7. One section was taken from seven mice on Day 14. Scale bar = 50 μm. Data are expressed as individual and mean ± SEM. * *p* < 0.05, ** *p* < 0.01 (two-way ANOVA followed by Sidak’s correction for multiple comparisons).

Increased Iba1 immunofluorescence in the IoN following dIoN-CCI was significantly attenuated by early perineural treatments of anti-HMGB1 nAb (Figure 6A,B; Two-way ANOVA, Sidak’s multiple comparisons test; Table S1).

#### 2.5.3. Microglia Activation

**Figure 7:**
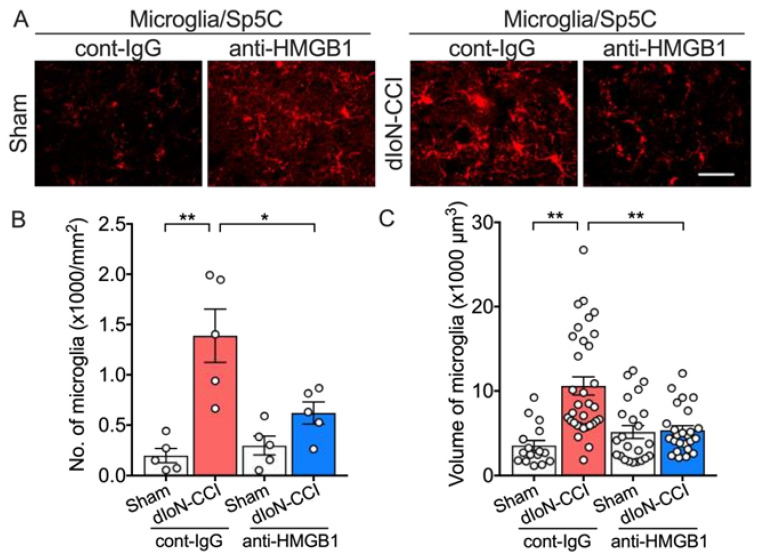
Effect of anti-HMGB1 nAb treatment on microglia in Sp5C in sham and dIoN-CCI mice. (**A**) Fluorescent photomicrographs of Iba1 in Sp5C from sham and dIoN-CCI mice 14 days following surgery. (**B**) density (number of cells/mm^2^) of *n* = 5 mice and (**C**) mean cell volume (*n* **=** 17–33 cells from 3 consecutive ipsilateral sections) 14 days following surgery. Scale bar = 50 μm. Data are expressed as individual and mean ± SEM. * *p* < 0.05, ** *p* < 0.01 (two-way ANOVA followed by Sidak’s correction for multiple comparisons).

Moreover, increased microglia number and increased microglia volume in the Sp5c were also significantly attenuated (Figure 7A–C; Two-way ANOVA, Sidak’s multiple comparisons test; Table S1). There were no changes in microglial cell volume and microglia number in the contralateral Sp5C (Figure S2; Two-way ANOVA, Sidak’s multiple comparisons test; Table S1).

## 3. Discussion

The current findings demonstrated that dIoN-CCI in mice evoked significant neuropathic pain-related behaviors, including increased facial grooming and sensitivity to a cooling stimulus. Following dIoN-CCI, mice developed a change in affect along with pain as suggested by preference to analgesic mirogabalin treatment over no treatment. Nerve injury also induced robust macrophage accumulation around the ipsilateral IoN and microglia activation in the ipsilateral Sp5C. Early perineural pretreatment with anti-HMGB1 nAb prevented the emergence of both neuropathic pain and immune cell activation, suggesting a key role of HMGB1 in the initiation of immune cell activation and pain related behaviors following nerve injury. 

While guidelines for the management of PTTN recommends drugs that are currently used for the management of neuropathic pain in general, such as duloxetine or pregabalin [16,17], some PTTN patients are not responsive to currently available treatments [18]. Perhaps with greater understanding of the mechanism of PTTN pain, more effective treatments can be developed. Peripheral nerve injury in rodents and primates evokes pain and ipsilateral cutaneous sensitivity to mechanical and cold stimuli [19,20]. Similarly, patients with a trigeminal nerve injury show long lasting mechanical/thermal hypersensitivity [21]. The current mouse model of dIoN-CCI was utilized to mimic clinical symptoms of PTTN. Following dIoN-CCI, mice displayed sensitivity to a cooling stimulus and spontaneous pain limited to the ipsilateral face. 

Furthermore, the current study showed that dIoN-CCI mice developed a change in pain-related affect, whereas sham mice did not, suggested by the preference to analgesic treatment over no treatment [22,23]. The effect of mirogabalin was likely due to its effect specifically on pain perception rather than potential reinforcing properties it may have as observed with opioids. Actually, gabapentinoids do not appear to induce preference in sham-operated mice [24]. In other rodent models of neuropathic pain, antinociceptive treatment induces CPP as well as reductions in nerve injury-induced pain-related behavior [25,26]. While not tested in the current study, it is possible that early blocking of peripherally expressed HMGB1 could prevent the onset of changed affect in neuropathic mice. Blocking peripheral afferents activated by inflammation also induces CPP [27]. Blocking changes in pain-associated affect by a peripheral mechanism, such as blocking HMGB1, could be a useful alternative to centrally acting pharmacotherapeutics.

Activation of peripheral and central immune cells is a key step in the induction and maintenance of chronic pain. Macrophage accumulate around the injured nerve and release inflammatory cytokines which leads to sensitization and activation of sensory nerves and to macrophage recruitment [28]. Activated microglia produce pro-inflammatory mediators which by themselves sensitizes neurons and leads to further activation of glia [13,29,30]. At the same time, both inhibition of macrophage accumulation on the sciatic nerve and injury-induced CNS microglial activation ameliorate cutaneous hypersensitivity [12,31]. Thus, blocking peripheral and central immune cell activation could lead to significant pain relief.

Extracellular HMGB1 appears to be involved in a number of inflammatory processes through receptors such as TLRs, RAGE and CXCR4 and play critical roles in the emergence and maintenance of neuropathic pain [32]. HMGB1 has been quantified in rodent models of painful peripheral neuropathies [33] including PTTN. HMGB1 is upregulated in the ipsilateral medulla oblongata after partial infraorbital nerve transection. Blocking HMGB1 in peripheral nerve injury models reduces both immune cell activation, macrophage recruitment and ipsilateral spinal cord glial activation, and ameliorates pain related behavior [8,10,34]. Intracisternal treatment with anti-HMGB1 nAb ameliorated injury-induced thermal hypersensitivity [14]. Whether the antinociceptive effect of cisternal anti-HMGB1 nAb treatment was accompanied by a reduction in glia activation was not reported and the effect of early vs. late treatment was not assessed. The current study demonstrated an association between reduced glia activation and pain relief by blocking peripherally expressed HMGB1. Furthermore, the current study suggests that HMGB1 appears to be mainly involved in the initiation of PTTN and further suggests that other factors, downstream of HMGB1, are involved in the activation of glia and the maintenance of PTTN. 

The current study demonstrated that early blocking of extracellular HMGB1 prevented the emergence of neuropathic pain, probably through the prevention of macrophage accumulation and microglia activation induced by nerve injury. The current findings confirm previous findings in other rodent models of neuropathic pain, in that HMGB1 is involved in the initiation but not the maintenance of neuropathic pain. As it is possible to assess the potential of developing PTTN pain, perhaps perioperative or early treatment with anti-HMGB1 could be utilized to prevent the onset of PTTN pain. In addition, given that the current study provides a new therapeutic candidate for PTTN prevention, further investigation to discovery new small molecules which can block HMGB1 receptors such as TLRs, RAGE, and CXCR4 is necessary.

## 4. Materials and Methods

### 4.1. Animals

Male ddY mice (RRID: MGI: 5652658; 33–38 g) were obtained from Japan SLC, Inc. (Shizuoka, Japan) at 5 weeks of age prior to infraorbital nerve surgery. These mice were established by the National Institute of Infectious Diseases (Tokyo, Japan) and have been used to model neuropathic pain [8,9,10]. Mice were housed in groups of five per cage with Clea paper bedding (CLEA Japan Inc., Tokyo, Japan). Cages were filter ventilated and within a sanitary barrier room. The room temperature was maintained at 22 ± 2 °C and humidity was 60 ± 5%. Lights were on a 12 h light/dark cycle (lights on/off at 8:00 a.m./8:00 p.m.). Mice before and after surgery were allowed free access to food (standard rodent chow; Oriental Yeast Co., Ltd., Osaka, Japan) and water ad libitum. Report on water purity was obtained from the Prefectural government once a year. All experiments utilizing animals were conducted in accordance with the “Guidelines for the Care and Use of Laboratory Animals” established by the Japanese Pharmacological Society. Procedures were reviewed and approved by the Committee of Research Facilities for Laboratory Animal Science of Hiroshima University (A16-94). All experiments were conducted by experimenters who were blind to the treatment condition of the animals. 

### 4.2. Distal Infraorbital Nerve Chronic Constriction Injury (dIoN-CCI) Model of Trigeminal Neuropathic Pain in Mice

A total of 142 mice were used. The dIoN-CCI surgery was performed following a procedure described previously [35]. Mice were anesthetized with 2% isoflurane in air and sodium pentobarbital (50 mg/kg, i.p.). Mice kept warm during surgery by placement on a heating pad. For aseptic technique, 10% povidone iodine was gently painted to the skin, and all surgical instruments were disinfected with 0.5% chlorhexidine prior to use and in between surgeries. Following preparation of the skin and clipping of the hair, a 3–4 mm skin incision was made between the left eye and whisker pad. The superficial fascia was gently separated, and the left IoN was exposed. Two 3-0 silk suture ligatures were loosely tied around the IoN about 1 mm apart. The incision was closed using one 6-0 silk suture. In sham-operated mice, the IoN was exposed but not ligated. Following the end of surgery, mice were placed in a warmed cage and observed for recovery from anesthesia. Each mouse was randomly chosen before dIoN-CCI or sham surgery.

### 4.3. Behavioral Assessments

Facial grooming time (recorded in seconds) was measured once in each mouse 7 days after surgery. Sensitivity to acetone were assessed 13 days after surgery (Figure S3A). Chambers were cleaned with 70% ethanol between mice. 

#### 4.3.1. Facial Grooming Time 

Mice were placed in a transparent Plexiglas chamber (6 × 9 × 18 cm) and habituated to the testing environment for 3 min. Episodes of ipsilateral facial grooming were counted over a period of 10 min. Facial grooming was defined as forelimb rubbing of the ipsilateral side of the face. The cumulative duration of grooming, in seconds, was recorded. 

#### 4.3.2. Acetone Test

Mice were placed in a transparent Plexiglas chamber (12 × 12 × 20 cm) and habituated to the chamber for 15 min. Acetone (15 µL) was dropped on the left whisker pad with a plastic micropipette with a polypropylene tip and the time spent rubbing its face, in seconds, over a period of 60 s. was recorded. Behaviors such as touching and rubbing the ipsilateral side of the face were counted as responses. A cut off of 60 s. was assigned if the mice did not respond to acetone. Each mouse was tested three separate times and the mean was reported. 

### 4.4. Conditioned Place Preference (CPP) 

The conditioned place preference test was used to indirectly assess for pain-related change in affect [26]. Preference to a specific place that is paired with analgesic treatment suggests a change in affect related to pain. The gabapentinoid mirogabalin (2 mg/kg, 10 mL/kg, i.p. (Daiichi Sankyo, Tokyo, Japan)) in saline, efficacious in mouse models of neuropathic pain and clinically efficacious in patients with neuropathic pain [36], was paired with one of the chambers to induce place preference. The dose of mirogabalin in the current study were based in part on previous study [37]. The shuttle box was composed of two equal-sized compartments (15 × 24 × 30 cm) with distinct tactile and visual cues (one compartment had a black wall with a gray smooth floor and the other compartment was white with a sandpaper-textured floor (Taiko Syokai, Hiroshima, Japan) [38]. The two chambers were separated by a removable door. Each CPP test consisted of a pre-conditioning test day (Day 1), conditioning days (Day 2–5), and a post-conditioned day (Day 6). Treatment schedule is illustrated in Figure S3B. On the pre-conditioning day, mice were placed in the shuttle box with the door open and the total time spent in each chamber was recorded (in sec.) over a 15 min. period. During the conditioning days, mice were treated with either mirogabalin or saline and placed in the chamber in it which spent the least amount of time (either the black or white chamber) for 45 min. Mice were dosed with either saline or mirogabalin at each session about 8 h apart from Day 2–5. The post-conditioning day was performed similarly to that during pre-conditioning days. Scores were calculated by subtracting the time spent in the mirogabalin-paired compartment during the pretest session from that spent in the same compartment during the test session: CPP score = (Day 6 preconditioning time (sec.)—Day 1 post-conditioning time (sec.)). Mice that did not move and remained in either chamber for more than 12 min before the conditioning day were not used in this study. Three non-operated mice were excluded from the study. 

### 4.5. Drug Treatment

A perioperative/early treatment schedule was used in one group of mice (Figure S3C). Anti-HMGB1 nAb (antibody no. 10–22, subclass IgG2a; antibody no. 4-1, subclass IgG2a, 100 ng, 10 µL) or an equal volume of control IgG (rat subclass IgG2a; anti-keyhole limpet hemocyanin) was applied directly to infraorbital nerve exposed during dIoN-CCI surgery using a plastic micropipette. Two days after IoN surgery, mice were anesthetized under 2% isoflurane, and either anti-HMGB1 (100 ng, 50 µL) or an equal volume of control IgG was infused around the IoN using a 1 mL syringe with a 29-gauge needle. Both antibodies were produced as described previously [39]. The antibodies (IgG) do not cross the blood- brain barrier [40]. The dose of anti-HMGB1 nAb used in the current study was based in part on previous studies [8,9,10,41]. In a separate group of mice, to test if anti-HMGB1 nAb treatment relieves pain-related behavior after nerve injury, mice were anesthetized under 2% isoflurane and treated with either anti-HMGB1 nAb (100 ng, 50 µL) or an equal volume of control IgG 7 and 9 days after nerve injury (Figure S2A).

### 4.6. Immunohistochemistry

Experiment schedules are illustrated in Figure S3C,D. Previous studies have demonstrated that peripheral nerve injury increases the number and size of microglia, indicating microglial activation, in spinal dorsal horn, [7,42]. Hence, in the current study, the number and morphological changes of microglia in trigeminal nucleus caudalis (Sp5C), wherein the central terminal of trigeminal nerves synapse with brainstem neurons, were examined. Mice were transcardially perfused under anesthesia with 2% isoflurane in air and sodium pentobarbital (50 mg/kg, i.p), with 50 mL saline followed by 25 mL of 4% (*w/v*) paraformaldehyde in 0.1 M phosphate buffer (pH 7.4). The IoNs and brains were quickly removed and post-fixed in 0.1 M phosphate-buffered 4% paraformaldehyde for two days at 4 °C and then cryoprotected for two days in 30% (*w/v*) sucrose in 0.1 M phosphate buffer at 4 °C. Brains were embedded in Shandon M-1 Embedding Matrix^™^ (Catalog # 1310TS; Thermo Fisher Scientific, Waltham, MA, USA) and frozen in liquid nitrogen. The spinal trigeminal nucleus caudalis and ipsilateral infraorbital nerve were cut serially (30 μm thickness) in a cryostat. Sections were collected onto glass slides. The sections were rinsed with phosphate-buffered saline, incubated in a blocking solution of 10% goat serum, 3% bovine serum albumin, 0.1% Triton-X and 0.05% Tween-20 in phosphate-buffered saline for 2 h at room temperature, and then incubated with primary antibodies: rabbit polyclonal anti-ionized calcium-binding adapter molecule 1 (Iba1, a marker for both macrophage and microglia) antibody (Catalog # 019-19741; RRID: AB_839504; 1:500; WAKO Pure Chemical Industries, Osaka, Japan). Sections were incubated in primary antibody for 2 days at 4 °C, followed by incubation in corresponding secondary antibodies with Alexa Fluor® 555 (Catalog # A21429, 1:500; Thermo Fisher Scientific) for 2 h at 4 °C in a dark chamber. The sections were then extensively washed in phosphate-buffered saline. Cover-slipped sections were examined with a BZ-9000 Biorevo all-in-one fluorescence microscope (Keyence, Elmwood Park, NJ, USA). Macrophage density in infraorbital nerve was quantified by measuring Iba1 intensity. Individual microglia cell volume was measured using 3D morph script [43]. In brief, RGB images were converted to gray scale and the volume of each microglia found in each image was measured. Images obtained from three mice were used for all 3D Morph experiences. One section was randomly selected from the Sp5C and IoN of each mouse. Within an area of 0.385 mm^2^, the number of cells were counted, microglia cell volume was measured, and microglia and macrophage cell fluorescence intensity were measured. All immunohistochemical experiments and analyses were performed in a blinded manner by an operator who was unaware of the treatment status of the mice from which the sections were taken.

### 4.7. Statistical Analysis

All mice that underwent either sham or dIoN-CCI surgery were used. Following collection of the data, the ROUT test (Q = 10%) was utilized to identify and exclude statistical outliers. Based on statistical testing, a total of 5 sham and 2 dIoN-CCI were identified as statistical outlies and excluded from data analysis. Following identification and removal of outliers, the data distribution was determined with D’Agostino & Pearson for experiments using 10 or more mice. The Shapiro–Wilk normality test was used in experiments using fewer than 10 mice. Using either test, data were found to be normally distributed. Therefore, comparisons between sham and dIoN-CCI treated mice were analyzed using an unpaired *t* test. Statistical analysis between pre- and post-conditioned tests was performed with a two-way repeated measures (RM) analysis of variance (ANOVA) followed by Sidak’s correction for multiple comparisons. To compare between sham and dIoN-CCI CCP scores, differences from 0 (i.e., no preference) were determined for each group using a one-sample *t* test as previously described [44]. To compare between the effect of anti-HMGB1 nAb and control IgG treatment, data were analyzed via a two-way ANOVA followed by Sidak’s correction for multiple comparisons. Individual data and group means (±SEM) are presented. Statistical analyses were performed with Prism 7.0e (GraphPad Software Inc., San Diego, CA, USA). The minimum level of statistical significant was *p* value less than 0.05. Detailed statistical results are summarized in Table S1.

## Data Availability

The data that support the findings of this study are available from the corresponding author, Norimitsu Morioka, upon reasonable request.

## Supplementary Materials

Figure S1: Effect of anti-HMGB1 nAb treatment after dIoN-CCI, Figure S2: Effect of anti-HMGB1 nAb treatment on the contralateral Sp5C following dIoN-CCI, Figure S3: Experimental schedule, Table S1: Summary of statistical analysis.
