# Supplementary material for: Pretreatment with High Mobility Group Box-1 Monoclonal Antibody Prevents the Onset of Trigeminal Neuropathy in Mice with a Distal Infraorbital Nerve Chronic Constriction Injury
Source: Molecules. 2021 Apr 2;26(7):2035. doi: 10.3390/molecules26072035 (PMC8038245; doi:10.3390/molecules26072035)
Supplement: Supplementary file 1 [file molecules-26-02035-s001.zip › Supplementary files/Supplementary Figure Legends (Molecules).docx]

**Figure S1.** Effect of anti-HMGB1 nAb treatment after dIoN-CCI.

(A) study time course. At 7 and 9 days after dIoN-CCI surgery (arrows), mice were treated with either control IgG or anti-HMGB1 nAb antibody. (B) responses to cooling stimulation (n = 10 mice per group) 13 days after dIoN-CCI surgery. There was no significant difference in response latency between control IgG and anti-HMGB1 nAb treatments (Unpaired *t* test). Data expressed as individual and group mean ± SEM.

**Figure S2.** Effect of anti-HMGB1 nAb treatment on the contralateral Sp5C following dIoN-CCI. Fluorescent photomicrographs of microglia from contralateral side Sp5C of sham and dIoN-CCI mice 14 days following surgery. Mean density (number/mm2) (n = 5 mice) of microglia in 14 days following surgery. Data expressed as individual and group mean ± SEM.. *p < 0.05, **p < 0.01 (two-way ANOVA followed by Sidak's multiple comparisons test).

**Figure S3.** Experimental schedule.

(A) Facial grooming time (reported in seconds) was measured once in each mouse 7 days after surgery. Sensitivity to acetone was assessed 13 days after surgery. (B) In the CCP test, the pre-conditioning test day was on Day 9, conditioning days were Day 10-13, and the post-conditioned day was Day 14. (C) Mice were treated perineurally with antibody, immediately and then two days after IoN-CCI surgery. Arrows indicate the time of treatment with either control IgG or anti-HMGB1 nAb. (D) Mice were perfusion fixed and processed for immunohistochemistry on Days 3, 7, and 14.
