# Supplementary figures and images for: Pretreatment with High Mobility Group Box-1 Monoclonal Antibody Prevents the Onset of Trigeminal Neuropathy in Mice with a Distal Infraorbital Nerve Chronic Constriction Injury
Source: Molecules. 2021 Apr 2;26(7):2035. doi: 10.3390/molecules26072035 (PMC8038245; doi:10.3390/molecules26072035)

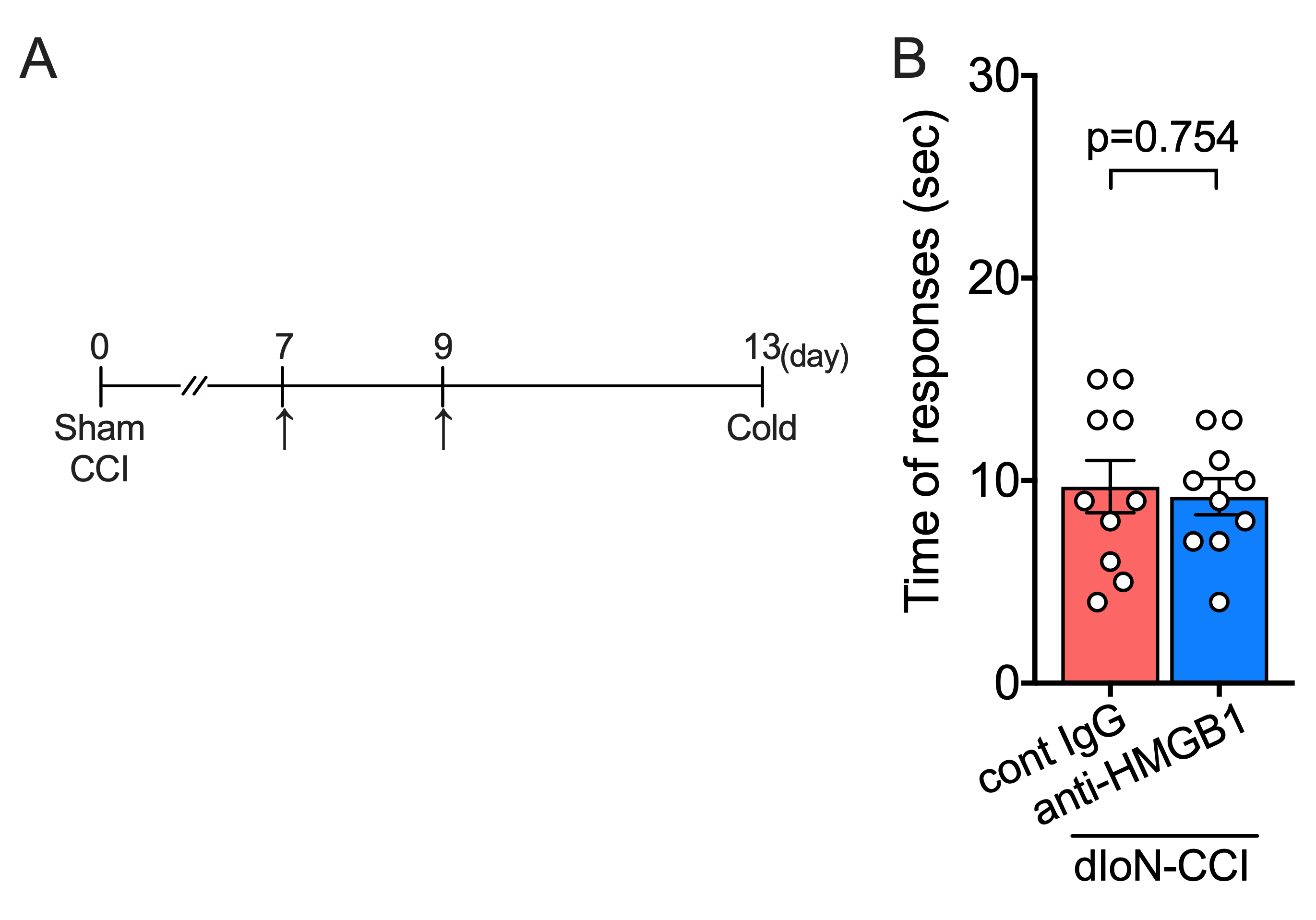

Supplement: Supplementary file 1 [file molecules-26-02035-s001.zip › Supplementary files/Fig. S1.tiff]

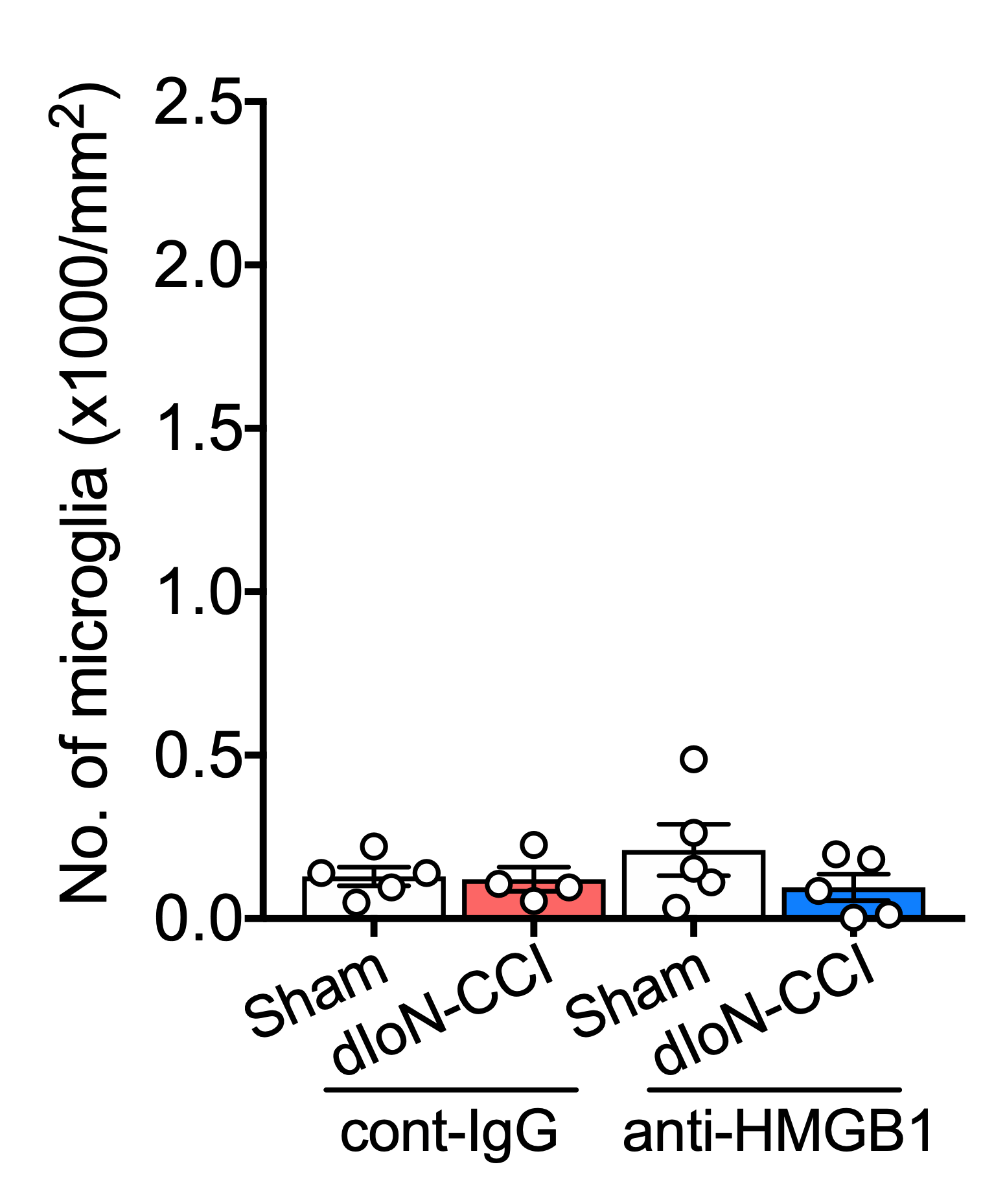

Supplement: Supplementary file 1 [file molecules-26-02035-s001.zip › Supplementary files/Fig. S2.tiff]

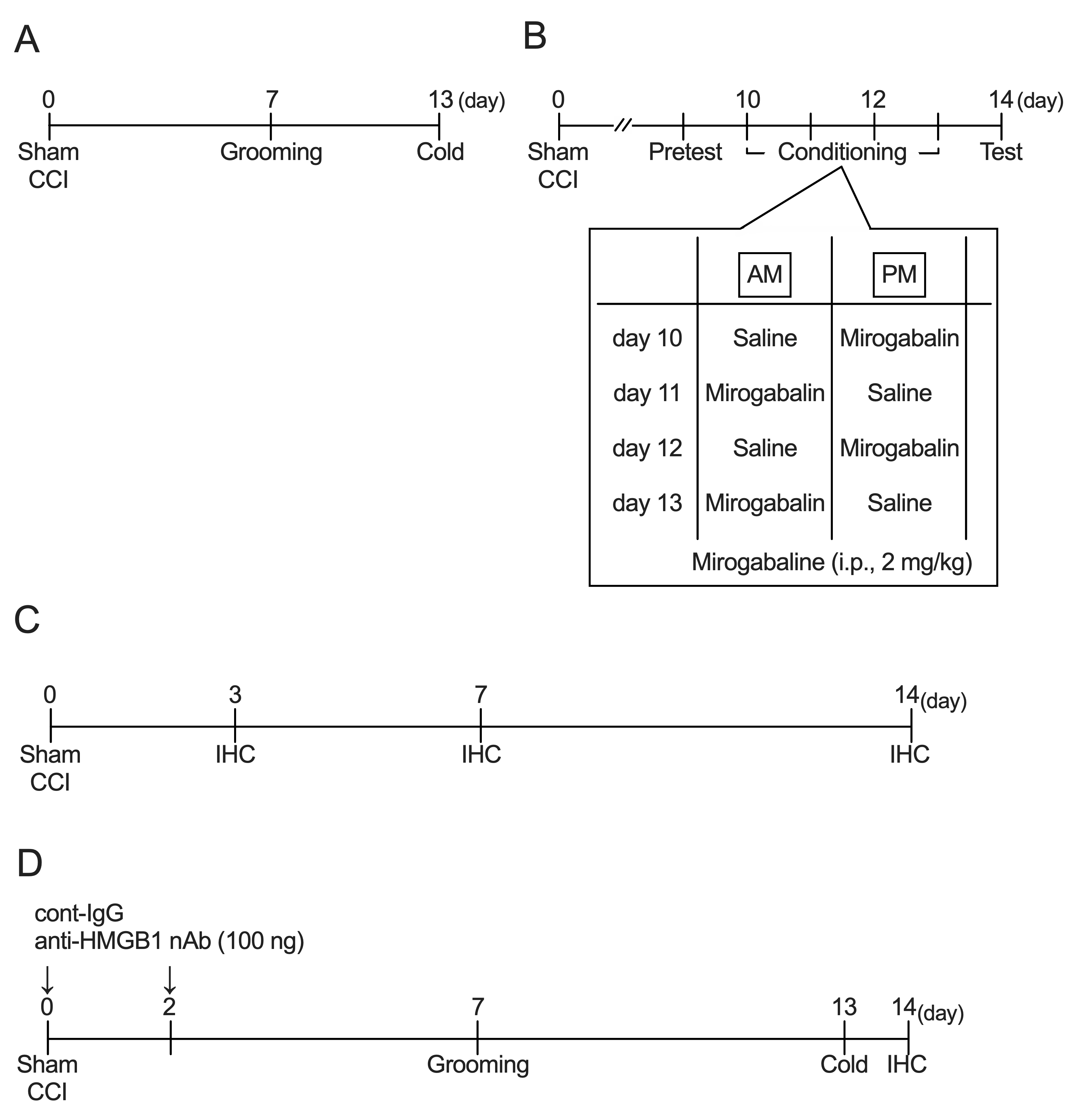

Supplement: Supplementary file 1 [file molecules-26-02035-s001.zip › Supplementary files/Fig. S3.tiff]
